# Comparison of the ability of newly inflammatory markers to predict complicated appendicitis

**DOI:** 10.1515/med-2024-1002

**Published:** 2024-07-25

**Authors:** Ali Saridas, Nafis Vural, Murat Duyan, Hasan Can Guven, Elif Ertas, Basar Cander

**Affiliations:** Department of Emergency Medicine, Prof. Dr. Cemil Tascioglu City Hospital, Istanbul, Turkey; School of Medicine, Bulent Ecevit University, Zonguldak, Turkey; Department of Emergency Medicine, Antalya Training and Research Hospital, Antalya, Turkey; Department of Emergency Medicine, Antalya Training and Research Hospital, Antalya, Turkey; Department of Biostatistics, Selcuk University, Konya, Turkey; Department of Emergency Medicine, Bezmialem Vakif University, Istanbul, Turkey

**Keywords:** acute appendicitis, complicated appendicitis, HALP, m-HALP, emergency department

## Abstract

**Introduction:**

Acute appendicitis (AA) is the predominant condition responsible for acute abdominal pain across all age demographics. The purpose of this research is to determine if the hemoglobin, albumin, lymphocyte, and platelet (HALP) and modified HALP (m-HALP) scores differ between complicated and uncomplicated appendicitis in patients diagnosed with AA who have applied to the emergency department (ED). Additionally, this study aims to investigate whether HALP and m-HALP scores are superior to other biomarkers.

**Materials and methods:**

The retrospective analysis included adult patients, aged eighteen or older, who were diagnosed with AA, and sought treatment at the ED of a tertiary hospital. Patients were divided into two groups: complicated appendicitis (CA) and uncomplicated appendicitis (UCA). The cut-off in diagnostic value measurements was determined using the receiver operating characteristic analysis.

**Results:**

A total of 436 patients (CA: 126, UCA: 310) were included. Neutrophil-to-lymphocyte ratio (NLR), neutrophil-to-albumin ratio, systemic immune-inflammation index (SII), systemic inflammation response index (SIRI), and pan-immune inflammation value (PIV) were found to have acceptable diagnostic power in CA detection (area under the curve [AUC]: 0.735–0.783). In detecting UCA, HALP and m-HALP were of fair diagnostic power (AUC: 0.64, 0.68, respectively).

**Conclusions:**

In this study, we found that although PIV, SIRI, SII, and NLR had acceptable diagnostic values in distinguishing CA and UCA, HALP and m-HALP had fair diagnostic values.

## 1 Introduction

The incidence rate of acute abdominal cases in emergency admissions ranges from 5 to 10% [1]. Acute appendicitis (AA) is the predominant condition responsible for acute abdominal pain across all age demographics [2]. AA may result in complications if there are delays in diagnosis. Complications can result in higher rates of morbidity and mortality [3]. AA is categorized into two groups: complicated appendicitis (CA) and uncomplicated appendicitis (UCA). Clinical complications such as abscess, gangrenous appendicitis, perforation, and phlegm are referred to as CA [4]. It is critical to ascertain whether the administration of AA is complicated.

Complete blood count (CBC) is a commonly utilized diagnostic test for AA, alongside any physical examination findings. Additionally, white blood cell count and neutrophil count are among the early indicators of inflammatory pathologies [5]. Neutrophil-to-lymphocyte ratio (NLR), platelet (PLT)/lymphocyte ratio, and systemic immune-inflammation index (SII) are inflammatory markers that can effectively differentiate between uncomplicated and complicated AA [6].

The hemoglobin, albumin, lymphocyte, and platelet (HALP) score is a comprehensive measure that includes hemoglobin, albumin, lymphocyte, and PLT levels. It has been recognized as a novel biomarker that can indicate both systemic inflammation and nutritional status [7]. The HALP score was identified as a dependable adverse biomarker for survival in a comprehensive meta-analysis [8]. Furthermore, HALP and modified HALP (m-HALP) scores have been identified as valuable indicators for predicting the outcome of acute cerebral ischemia and acute heart failure [9,10]. A recent study proposed that the HALP score can be utilized to forecast the severity and extent of complications associated with AA [11].

The purpose of this research is to determine whether a difference between the HALP and m-HALP exists between complicated and UCA in patients diagnosed with AA who have applied to the emergency department (ED). Additionally, this study aims to investigate whether HALP and m-HALP scores are more reliable compared to other biomarkers.

## 2 Materials and methods

### 2.1 Study design and settings

The retrospective cross-sectional study design consisted of adult patients aged 18 and over who were diagnosed with AA and sought treatment in the ED of a tertiary hospital between January 1, 2020, and January 1, 2023. The study received approval, and the requirement for informed consent was waived by the Ethics Commission (ethics committee decision number: 2023/243 date: November 20, 2023). The research was conducted in accordance with the Declaration of Helsinki.

### 2.2 Study protocol

After the ethics committee approval was received, the data on the hospital’s data network were retrospectively examined for patients diagnosed with AA aged 18 years and above. Demographic data (gender, age, etc.), laboratory findings, surgical operation notes, pathology reports, and abdominal CT results at the time of first admission to the ED were evaluated.

Patients who were either pregnant, had peripheral vascular disease, had previously suffered heart failure, had hematological disease, or had liver disease; were using anticoagulants, antibiotics, or steroids at time; had other acute or chronic infections; and had a pathology result revealing a tumor or whose records could not be accessed were all excluded from the study sample.

Patients who were diagnosed with AA based on a physical examination, ultrasonography, abdominal CT, and histopathology that underwent surgery were included in the study. The clinical condition of the patients, abdominal CT, surgery reports, and histopathological examinations of the appendix were evaluated en masse, and AA was classified as either complicated or uncomplicated. The clinical conditions and radiological data of the patients were not evaluated and analyzed individually. CA was defined as the presence of gangrenous or perforated appendicitis and/or diffuse peritonitis. UCA was defined as a phlegmatic inflamed appendix with no signs of necrosis or perforation [12]. Patients whose complicated or uncomplicated conditions could not be confirmed were excluded from the study sample.

### 2.3 Laboratory analyses

An automated hematology analyzer (Coulter Gen-S Hematology Analyzer; Beckman Coulter Corp, Hialeah, FL, USA) was used to determine the CBC. Hematological parameters, such as total leucocyte counts and differential, hemoglobin, hematocrit, PLT levels, NLR, neutrophil-to-albumin ratio (NAR), SII, systemic inflammation response index (SIRI), and pan-immune inflammation value (PIV), were recorded.

Serum sodium, potassium, glucose, urea, creatinine, albumin, alanine aminotransferase (ALT), aspartate aminotransferase (AST), and C-reactive protein (CRP) levels were recorded.

The NLR, NAR, PIV, SII, and SIRI were defined as “neutrophil count/lymphocyte count,” “neutrophil count/albumin level,” “neutrophil count × platelet count × monocyte count/lymphocyte count,” “neutrophil count × platelet count/lymphocyte count,” and “neutrophil × monocyte/lymphocyte count,” respectively. The HALP score was calculated by using the hemoglobin (g/L) × albumin (g/L) × lymphocyte count (1/L)/PLT count (1/L) method. The modified HALP score was calculated by using the hemoglobin (g/L) × albumin (g/L) × lymphocyte count (1/L) × PLT count (1/L) method.

### 2.4 Data analysis

Parametric tests were used without the normality test due to the compatibility of the central limit theorem [13]. In the analysis of the data, the mean, standard deviation, and minimum and maximum values of the features were used while performing the statistics of continuous data. Categorical variables were defined using frequency and percentage values. Student’s *t*-test statistics were used to compare the CA and UCA patients. Chi-square test statistics were used to evaluate the relationship between the two independent categorical variables. The receiver operating characteristic (ROC) analysis was used to determine the cut-off value of NLR, NAR, SII, SIRI, PIV, HALP, and m-HALP in predicting CA and UCA. Cut-off accuracy was evaluated with sensitivity and specificity statistics. The area under the curve (AUC) of 0.5–0.6 was interpreted as poor, 0.6–0.7 as fair, 0.7–0.8 as acceptable, 0.8–0.9 as excellent, and >0.9 as outstanding. The level of statistical significance of the data is considered *p* < 0.05. New York software (e-picos, New York, NY, USA, www.e-picos.com) and the MedCalc statistical package program (MedCalc Software Ltd., Ostend, Belgium) were used for data evaluation.

## 3 Results

A total of 436 patients (CA: 126, UCA: 310) were included in the study. Table 1 shows the average and standard deviation values of age, gender, and biomarkers. There is a significant relationship between the study groups and gender (*p* < 0.05). While 64% of the CA patients were male, only 51% of the UCA patient group were male. There is also a significant relationship between the study groups and age of the patients. The mean age of CA patients was lower compared to UCA patients.

**Table 1:** Comparison of basic and laboratory characteristics of complicated acute appendicitis and uncomplicated acute appendicitis groups

	CA (*n* = 126)	UCA (*n* = 310)	*p*-value
	*x̄* ± SD	*x̄* ± SD	
Age (years)	35.14 ± 13.81	39.21 ± 31.09	<0.028*
	*n* (%)	*n* (%)	
**Sex**			
Female	45 (36)	152 (49)	0.02**
Male	81 (64)	158 (51)	
**Features**			
Glucose (mg/dL)	115.46 ± 31.09	109.37 ± 29.80	0.36*
Serum sodium (mEq/L)	138.41 ± 2.8	139.48 ± 3.02	0.65*
Serum potassium (mEq/L)	4.22 ± 0.35	4.21 ± 0.37	0.007*
Urea (mg/dL)	31.32 ± 12.93	28.37 ± 9.89	0.004*
Creatinine (mg/dL)	0.87 ± 0.31	0.75 ± 0.29	0.14*
AST (U/L)	22.32 ± 8.71	19.17 ± 7.49	0.06*
ALT (U/L)	29.92 ± 16.06	19.15 ± 10.05	0.001*
CRP (mg/L)	65.71 ± 43.64	18.48 ± 16.26	<0.001*
Albumin	39.74 ± 2.57	40.98 ± 3.23	0.003*
Hemoglobin	14.74 ± 1.79	14.11 ± 1.53	0.23*
HTC	43.01 ± 4.61	41.25 ± 4.51	0.72*
PLT (10^3^ μL)	263.91 ± 56.57	259.45 ± 58.16	0.021*
NEU (10^3^ μL)	11.73 ± 3.54	9.16 ± 2.81	0.008*
LYM (10^3^ μL)	1.73 ± 0.55	2.38 ± 0.86	<0.001*
MON (10^3^ μL)	1.01 ± 0.21	0.98 ± 0.26	<0.001*
NLR	7.31 ± 2.75	4.54 ± 2.68	<0.001*
NAR	0.31 ± 0.09	0.23 ± 0.07	<0.001*
SII	1886.54 ± 850.62	1218.21 ± 634.21	<0.001*
SIRI	7.19 ± 3.26	4.25 ± 3.03	<0.001*
PIV	1915.02 ± 973.79	1133.27 ± 741.13	<0.001*
HALP	4.18 ± 1.81	5.18 ± 2.39	0.001*
m-HALP	2776,941.82 ± 115867.7	394267.1 ± 189061.5	0.001*

*Student’s *t* test, **Chi-square test (*p* < 0.05 significance).

AST: aspartate aminotransferase, ALT: alanine aminotransferase, CRP: C-reactive protein, INR: international normalized ratio, PLT: platelets, NEU: neutrophil, LYM: lymphocyte, MON: monocyte, NLR: neutrophil-to-lymphocyte ratio, SII: systemic immune inflammation index, SIRI: systemic inflammation response index, PIV: *pan*-immune inflammation value, HALP: hemoglobin × albumin levels × lymphocyte count/platelet count, m-HALP: modified HALP.

There was no significant difference between the group mean values of glucose, serum sodium, creatinine, AST, hemoglobin, and hematocrit (*p* > 0.05).

There was a significant difference between the group means of serum potassium, urea, ALT, CRP, albumin, PLT, neutrophil count (NEU), lymphocyte count (LYM), monocyte count (MON), NLR, NAR, SII, SIRI, PIV, HALP, and m-HALP values (*p* < 0.05) (Table 1).

**Figure 1:**
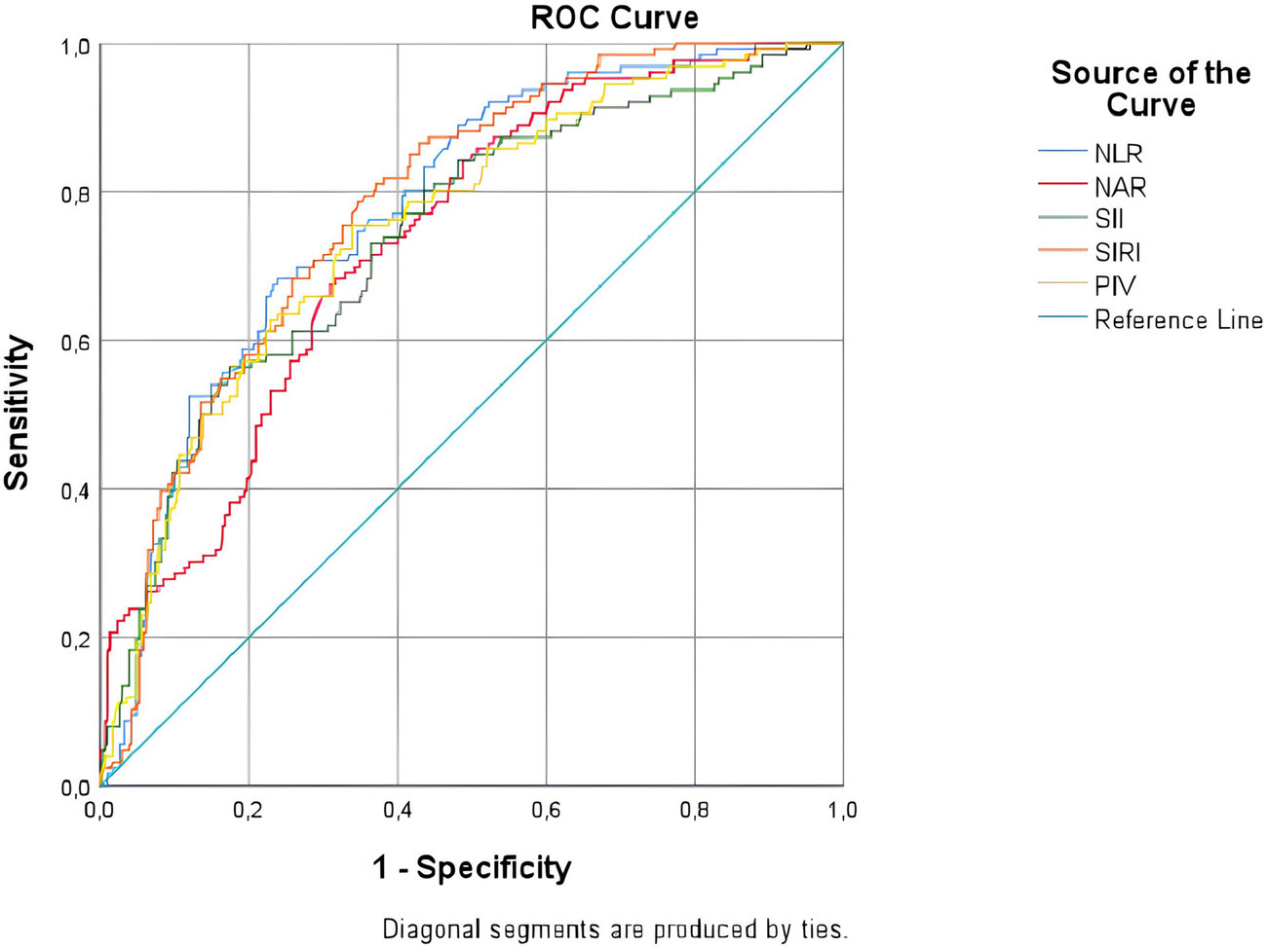
The ROC curves of biomarkers for predicting complicated acute appendicitis. NLR: neutrophil-to-lymphocyte ratio, NAR: neutrophil-to-albumin ratio, NPR: neutrophil-to-platelet ratio, SII: systemic immune inflammation index, SIRI: systemic inflammation response index, PIV: *pan*-immune inflammation value.

**Figure 2:**
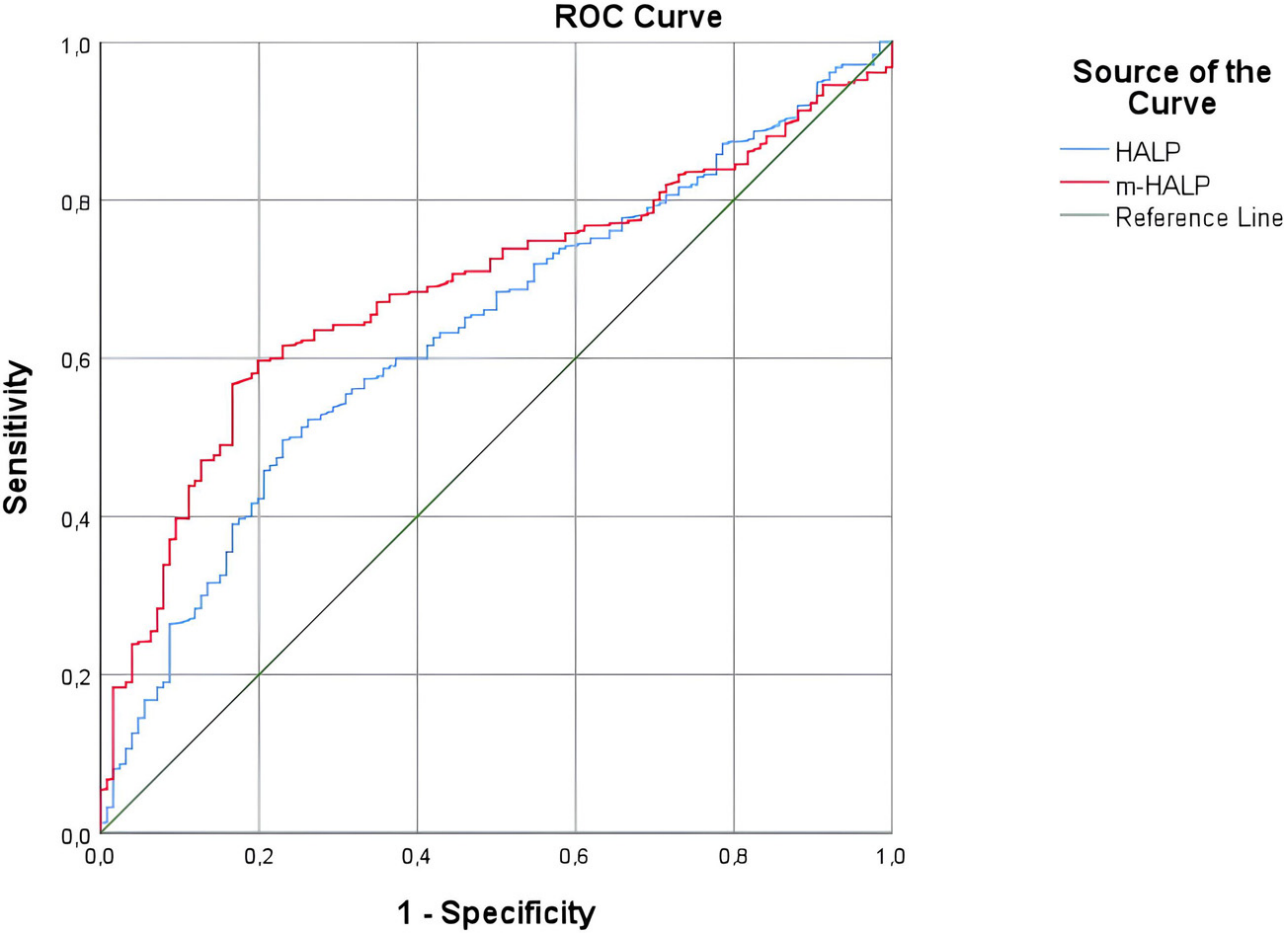
The ROC curves of biomarkers for predicting uncomplicated acute appendicitis. HALP: hemoglobin × albumin levels × lymphocyte count/platelet count, m-HALP: modified HALP.

Table 2 presents in detail the diagnostic accuracy of biomarkers important for the differential diagnosis of CA and UCA in ROC analysis (Tables 2 and 3, Figures 1 and 2).

**Table 2:** Diagnostic accuracy of inflammatory parameters to predicting complicated acute appendicitis

CA: 126	AUC	Cut-off	Sensitivity %	Specificity %	AUC 95% CI	*p*-value
UCA: 310						
NLR	0.778	>5.43	68.3	76.1	0.74–0.82	<0.001
NAR	0.735	>0.23	68.7	68.4	0.69–0.78	<0.001
SII	0.742	>1647.01	82.6	56.3	0.70–0.79	<0.001
SIRI	0.783	>4.45	78.6	65.5	0.74–0.82	<0.001
PIV	0.761	>1179.81	75.4	66.1	0.71–0.79	<0.001

AUC, area under curve; SE, standard error; PPV, positive predictive value; NPV, negative predictive value; CI, confidence interval; NLR, neutrophil-to-lymphocyte ratio; NAR, neutrophil-to-albumin ratio; NPR, neutrophil-to-platelet ratio, SII: systemic immune inflammation index, SIRI: systemic inflammation response index, PIV: *pan*-immune inflammation value.

**Table 3:** Diagnostic accuracy of inflammatory parameters to predicting uncomplicated acute appendicitis

CA: 126	AUC	Cut-off	Sensitivity %	Specificity %	AUC 95% CI	*p*-value
UCA: 310						
HALP	0.64	>4.69	77.2	51.8	0.59–0.68	<0.001
m-HALP	0.68	>348,737.98	83.3	56.8	0.64–0.73	<0.001

HALP: hemoglobin × albumin levels × lymphocyte count/platelet count m-HALP: The modified HALP.

NLR, NAR, SII, SIRI, and PIV were found to have acceptable diagnostic power in CA detection (AUC: 0.735–0.783).

In detecting UCA, HALP and m-HALP were of fair diagnostic power (AUC: 0.64, 0.68, respectively).

## 4 Discussion

It is critical to distinguish whether AA is complicated or not, as well as to diagnose it correctly. While clinical studies analyze the diagnosis of AA itself, few have attempted to distinguish between CA and UCA [12]. However, the guidelines currently do not contain explicit instructions on how to differentiate between uncomplicated and complicated cases of appendicitis [14,15]. The same guidelines recommend that CA should be treated more urgently and that UCA can only be treated with antibiotics [14,15]. Therefore, it is important to make a differential diagnosis of complications or UCA in a patient diagnosed with AA.

The constituent elements of the HALP score can offer crucial insights into the immunological nutritional state of the patient. Assessing hemoglobin levels is an important procedure because hemoglobin levels are an indicator of anemia, which is a prevalent illness worsened by many inflammatory processes. Albumin, which is a protein produced by the liver, is classified as a negative acute phase reactant. Low levels of albumin in the blood are indicative of an inflammatory condition. Lymphocytes and PLTs play critical roles in the immunological function of the body, decreased lymphocyte count, and elevated PLT count may suggest a compromised immune system with a heightened susceptibility to infection [16]. According to this data, low HALP and m-HALP scores are indicative of the intensity of inflammation. Therefore, low scores can be seen as an indirect indication of CA.

A study conducted after surgery found that a low HALP score was linked to complications and postoperative morbidity in patients of AA [11]. Patients diagnosed with metastatic stomach cancer exhibited significantly lower survival rates compared to individuals with high HALP scores [17]. A separate study demonstrated that a low HALP score can differentiate between malignant origins in patients experiencing extrahepatic biliary obstruction [18]. A meta-analysis conducted on cancer patients has determined that a low HALP score can serve as a negative biomarker for survival [8]. Since inflammation is more severe in patients with CA, albumin and lymphocyte levels are expected to decrease. Therefore, unlike other inflammatory biomarkers, the HALP and m-HALP scores are expected to be negative biomarkers. Inflammatory biomarkers such as PIV, SIRI, SII, and NLR are expected to be positive biomarkers in distinguishing complicated and uncomplicated patients with AA. Thus, when evaluating HALP and m-HALP scores, it is expected to be higher in UCA. In this study, HALP and m-HALP scores had a fair diagnostic value in distinguishing uncomplicated and complicated patients with AA.

Elevated NLR levels are associated with an increase in neutrophils or a decrease in lymphocytes during inflammation of the appendix [19]. Increased NLR levels are associated with an increase in neutrophils or a decrease in lymphocytes during inflammation of the appendix [20]. A recent similar study showed that NLR predicted CA with an AUC of 0.730 [21]. In this study, we found that NLR can predict CA with a higher diagnostic value.

SIRI and SII, which are usually assessed based on peripheral blood-based parameters (e.g., lymphocytes, CRP, monocytes, neutrophils, or PLT count), have been shown to be associated with several types of cancer [22,23]. A study undertaken throughout the COVID-19 pandemic revealed a correlation between SII and SIRI and the occurrence of AA complications [24]. In the study conducted by Tekeli et al., SII was found to be useful in determining CA. In this study, it was concluded that SII and SIRI could be useful in detecting complicated AA. This conclusion supports the existing literature.

PIV is a novel composite biomarker utilized to assess inflammatory conditions. Unlike current biomarkers, PIV incorporates all four primary cell types seen in peripheral blood [25]. A study conducted in patients with rheumatoid arthritis determined that PIV is an effective tool for differentiating between periods of active disease and times of remission [26]. Additionally, it has been discovered to be a promising biomarker in individuals diagnosed with colorectal cancer [27]. Nevertheless, the connection between AA and PIV remained ambiguous. This study demonstrated that the diagnostic efficacy of PIV in differentiating between difficult and UCA was comparable to that of previously used inflammatory indicators.

There is still no parameter with a high diagnostic value in distinguishing between complicated and uncomplicated AA patients. According to the results of our study, when compared to HALP and m-HALP scores, inflammatory biomarkers such as PIV, SIRI, SII, and NLR had a higher diagnostic value in making this distinction.

## 5 Study limitations

As our study was planned to be single-centered and retrospective, examination findings could not be obtained. The assessment of AA solely focused on determining whether it was complicated or not, without considering the surgical intervention or the length of hospitalization post-surgery. Since most of the patients diagnosed with AA were operated on in the hospital where the study was conducted, the number of those who were not operated on was low. These patients were not included in the study as they did not have a definitive histopathological diagnosis. The clinical condition of the patients, abdominal CT, surgery reports, and histopathological examinations of the appendix were evaluated collectively, and AA was classified as complicated or uncomplicated. The clinical conditions and radiological data of the patients were not evaluated and analyzed individually. These are the important limitations of our study. Large-scale prospective studies are needed for more reliable results.

## 6 Conclusions

The utilization of inflammatory biomarkers, derived from routine blood tests commonly employed in the diagnostic protocol within the ED, is more prevalent due to its ease of calculation and cost-effectiveness. In this study, we found that, although PIV, SIRI, SII, and NLR had acceptable diagnostic values in distinguishing complicated and uncomplicated AA, HALP and m-HALP also had a fair diagnostic value.
